# Pulmonary hypertension and lung transplantation waitlist outcomes for hypersensitivity pneumonitis

**DOI:** 10.1016/j.jhlto.2024.100157

**Published:** 2024-09-07

**Authors:** Michael J. Nicholson, Zehra Dhanani, Shameek Gayen

**Affiliations:** a Thoracic Medicine and Surgery, Temple University Hospital, Philadelphia, Pennsylvania; b Thoracic Medicine and Surgery, Lewis Katz School of Medicine at Temple University, Philadelphia, Pennsylvania

**Keywords:** alveolitis, extrinsic allergic, lung diseases, interstitial, pulmonary circulation

## Abstract

**Background:**

Hypersensitivity pneumonitis is an interstitial lung disease that can progress to pulmonary hypertension which increases mortality. The European Society of Cardiology recently reduced the diagnostic criteria for precapillary pulmonary hypertension from mean pulmonary artery pressure ≥25mmHg to ≥20mmHg and pulmonary vascular resistance from ≥3Wu to ≥2Wu.

**Methods:**

We conducted a retrospective cohort study of the Scientific Registry of Transplant Recipients.1175 hypersensitivity pneumonitis patients listed for lung transplantation were divided into three groups based on the presence of precapillary pulmonary hypertension; those with pre-capillary pulmonary hypertension based on old criteria, new criteria, and those without precapillary pulmonary hypertension based on either criterion. The individual components of precapillary pulmonary hypertension were also assessed with three mean pulmonary artery pressure groups (≤20mmHg, >20mmHg to <25mmHg, ≥25 mmHg) and three pulmonary vascular resistance groups (≤2Wu, >2Wu to <3Wu, ≥3Wu). Survival analysis was performed.

**Results:**

Kaplan-Meier analysis showed a difference in waitlist survival probability among the mean pulmonary artery pressure groups and among the pulmonary vascular resistance groups driven by the most severe groups. Multivariate Cox regression showed no difference between the various pre-capillary pulmonary hypertension groups. Pulmonary vascular resistance ≥3Wu was associated with increased waitlist mortality.

**Conclusions:**

The new thresholds of mean pulmonary artery pressure and pulmonary vascular resistance do not appear to prognosticate waitlist outcomes when combined. PVR ≥3Wu is associated with worse waitlist outcomes and may be useful in prognostication.

## Background

Hypersensitivity pneumonitis (HP) is an interstitial lung disease (ILD) characterized by pulmonary inflammation in response to repetitive antigen inhalation. Susceptible individuals experience an exaggerated immune reaction to inhaled antigens leading to a wide variety of parenchymal changes.^1^ The true prevalence of HP is uncertain due to a lack of standardized diagnostic criteria. Within the general population, there are estimated to be approximately 2 cases per 100,000 persons, and HP is estimated to comprise between 1.5% and 13% of all ILDs.^2^ While many cases can be resolved with the removal of the triggering antigen, those that progress pose a significant challenge, as no validated medical therapies exist.^1^, ^2^

The progression of HP may lead to worsening respiratory failure, necessitating evaluation for lung transplantation. Lung transplantation has demonstrated excellent short- and medium-term survival rates in HP. Studies indicate 1-, 3-, and 5-year survival rates as high as 96%, 89%, and 89%, respectively.^3^ Consequently, lung transplantation will continue to be an essential treatment modality for appropriately selected patients with refractory HP.

Accurate prognostication of HP is crucial for patients undergoing lung transplantation evaluation and listing. Group 3 pulmonary hypertension (PH) is a potential complication of all ILDs, including HP.^4^, ^5^ While the exact prevalence of PH in HP is unclear, it is reported to be significant and its presence increases morbidity and mortality.^2^, ^6^ This is reflected in the Lung Composite Allocation Score (L-CAS) which includes the severity of PH as a variable in its calculation. PH increases the L-CAS and, therefore, the patient’s priority for lung transplantation.^7^ This emphasizes the need for accurate diagnosis and classification of PH in lung transplantation candidates.

In 2022, the European Society of Cardiology (ESC) revised its diagnostic criteria for precapillary PH. Notably, they lowered the threshold for mean pulmonary artery pressure (mPAP) from 25 to 20 mm Hg and the pulmonary vascular resistance (PVR), from 3 to 2 Wood units (WU).^8^ These adjusted criteria broaden the population of patients considered to have precapillary PH. We hypothesize that patients now considered to have precapillary PH based on the new criteria would have higher waitlist mortality than those without PH. Our objective was to compare the waitlist outcomes of those with precapillary PH under the new diagnostic criteria to those without PH and those with PH under the old criteria.

## Materials and methods

### Approvals

This study was approved by the Institutional Review Board of Temple University Hospital. This study is in compliance with the International Society for Heart and Lung Transplantation Ethics Statement.

### Subjects

We conducted a retrospective cohort study of the Scientific Registry of Transplant Recipients (SRTR) examining patients listed for lung transplantation between January 1, 2000 and December 31, 2022. Our analysis was restricted to adult lung transplant candidates (≥18 years of age) with the primary diagnosis of HP. All patients undergoing lung transplantation evaluation undergo a right heart catheterization.

### Data collection

Baseline characteristics including age, sex, race, and body mass index (BMI) were collected. Clinical data, including forced vital capacity (FVC), blood type, hemodynamic data, mPAP, and PVR values, were collected. Patients were divided into 3 groups; no PH based on both criteria, PH based only on old criteria, and PH based on new criteria. The patients were also divided into 3 mPAP groups (≤20, >20 to <25, and ≥25 mm Hg) and 3 PVR groups (≤2, >2 to <3, and ≥3 WU).

The data reported here have been supplied by the Hennepin Healthcare Research Institute as the contractor for the SRTR. The interpretation and reporting of these data are the responsibility of the author(s) and in no way should be seen as an official policy of or interpretation by the SRTR or the U.S. Government. This study used data from the SRTR. The SRTR data system includes data on all donors, wait-listed candidates, and transplant recipients in the United States, submitted by the members of the Organ Procurement and Transplantation Network. The Health Resources and Services Administration, U.S. Department of Health and Human Services provides oversight to the activities of the Organ Procurement and Transplantation Network and SRTR contractors. The proposed study was approved by the SRTR.

### Statistical analysis

All continuous variables were presented as mean ± standard deviation or median (interquartile range) unless otherwise stated. The categorical variables were compared using Pearson’s chi-square test or Fisher’s exact test where applicable. The continuous variables were compared between groups using the Mann-Whitney U test.

#### Subjects

FVC percent predicted (% pred) mean values were collected for each of the mPAP and PVR groups. Analysis of variance (ANOVA) analysis was performed comparing FVC % pred between the mPAP groups and between the PVR groups.

#### Combined mean pulmonary arterial pressure and pulmonary vascular resistance

Multivariable Cox regression was performed between the 3 diagnostic groups. Pertinent variables, including FVC, 6-minute walk distance (6MWD), oxygen requirement, height, age, sex, BMI, and blood type, were accounted for.

#### Mean pulmonary arterial pressure

Survival analysis was performed via Kaplan-Meier analysis and Cox regression. For the investigation of mPAP, initial Kaplan-Meier analysis was performed on the 3 mPAP groups (≤20, >20 to <25, and ≥25 mm Hg). Repeat analysis was performed exclusively on the mPAP ≤20 mm Hg group and the 20 to 25 mm Hg group. Cox regression was performed to determine the association of the mPAP groups with waitlist mortality. The mPAP >20 to <25 mm Hg group and the mPAP ≥25 mm Hg group were compared to the mPAP ≤20 mm Hg group to determine the relative risk of waitlist mortality. Multivariable Cox regression was then performed between the aforementioned groups to account for pertinent variables, including FVC, 6MWD, oxygen requirement, height, age, sex, BMI, and blood type.

#### Pulmonary vascular resistance

Survival analysis was performed via Kaplan-Meier analysis and Cox regression. For the investigation of PVR, initial Kaplan-Meier analysis was performed between the 3 PVR groups (≤2, >2 to <3, and ≥3 WU). Repeat analysis was performed exclusively comparing the PVR ≤2 WU group to the >2 to <3 WU group. Cox regression was performed to determine the association of the PVR groups with waitlist mortality. The PVR >2 to <3 WU group and the PVR ≥3 WU groups were compared to the PVR ≤2 WU group to determine the relative risk of waitlist mortality. Multivariable Cox regression was then performed between the aforementioned groups to account for pertinent variables, including FVC, 6MWD, oxygen requirement, height, age, sex, BMI, and blood type.

## Results

### Subjects

**Figure 1:**
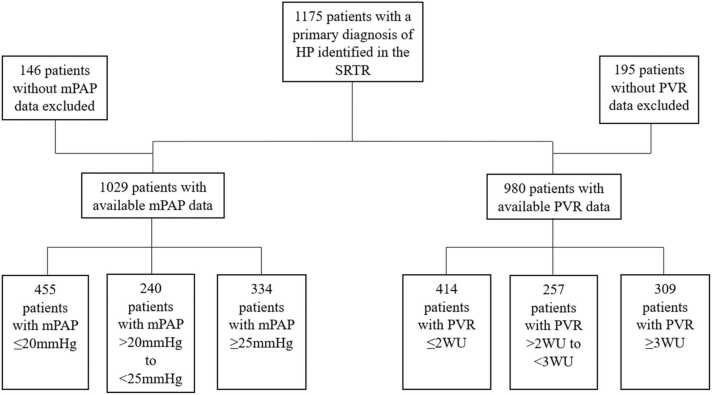
Distribution of HP patients into mPAP and PVR groups. HP, hypersensitivity pneumonitis; mPAP, mean pulmonary artery pressure; PVR, pulmonary vascular resistance; SRTR, Scientific Registry of Transplant Recipients; WU, Wood units.

We identified 1,175 patients listed for lung transplantation with the diagnosis of HP (Figure 1). The baseline demographic and clinical characteristics of this population are detailed in Table 1. Nine hundred and eighty patients had both mPAP and PVR data available. Of those 980 patients, 505 patients met the criteria for precapillary PH (mPAP >20 mm Hg, PVR ≥2 mm Hg, and pulmonary capillary wedge pressure ≤15) for a prevalence of 52%.

**Table 1:** Baseline Demographic and Clinical Characteristics of the Study Population

Characteristic	Subjects (*n* = 1,029)
Race, *n* (%)	
White	923 (89.7)
Black	43 (4.2)
Asian	56 (5.4)
Other	7 (0.7)
FVC,* % pred	40.3 ± 13.9
FEV1,† % pred	43.3 ± 14.8
6MWD,‡ m	238.9 ± 139.4
PASP,§ mm Hg	39.3 ± 14.1
mPAP,^ll^ mm Hg	22.7 ± 9.9
PCWP,** mm Hg	9.2 ± 5.2
CO,†† liter/min	5.5 ± 1.5
PVR,‡‡ WU	2.8 ± 2.3
Resting supplemental O_2_ requirement, liter/min	5.3 ± 5.1
Blood type, *n* (%)	
A	450 (38.3)
AB	54 (4.6)
B	139 (11.8)
O	532 (45.3)

Abbreviations: % pred, percent predicted; 6MWD, 6-minute walk distance; CO, cardiac output; FEV1, forced expiratory volume in 1 second; FVC, forced vital capacity; mPAP, mean pulmonary artery pressure; PCWP, Pulmonary capillary wedge pressure; PASP, pulmonary artery systolic pressure; WU, Wood units.

* Functional vital capacity.

† Forced expiratory volume in 1 second.

‡ Six-minute walk distance.

§ Pulmonary artery systolic pressure.

ll Mean pulmonary artery pressure.

** Pulmonary capillary wedge pressure

†† Cardiac output

‡‡ Pulmonary vascular resistance

### Lung function

Mean FVC % pred was 39.3%, 40.5%, and 41.8% for the mPAP ≤20 mm Hg, mPAP >20 to <25 mm Hg, and mPAP ≥25 mm Hg, groups, respectively. ANOVA analysis comparing FVC % pred across the 3 mPAP groups did not show a statistically significant difference between groups (*p* = 0.45) (Table 2). Mean FVC % pred was 39.7%, 41.6%, and 40.4% for the PVR ≤2 WU, PVR >2 to <3 WU, and PVR ≥3 WU, groups, respectively. ANOVA analysis comparing FVC % pred across the 3 PVR groups did not show a statistically significant difference between groups (*p* = 0.71) (Table 3).

**Table 2:** Mean FVC % Pred Between the mPAP Groups

mPAP group (mm Hg)	FVC, % pred	ANOVA
≤20	39.3	
>20 to <25	40.5	
≥25	41.8	*p* = 0.45

Abbreviations: % pred, percent predicted; ANOVA, Analysis of variance; FVC, forced vital capacity; mPAP, mean pulmonary artery pressure.

**Table 3:** Mean FVC % Pred Between the PVR Groups

PVR group (WU)	FVC, % pred	ANOVA
≤2	39.7	
>2 to <3	41.6	
≥3WU	40.4	*p* = 0.71

Abbreviations: % pred, percent predicted; ANOVA, Analysis of variance; FVC, forced vital capacity; PVR, pulmonary vascular resistance; WU, Wood units.

### Combined mean pulmonary artery pressure and pulmonary vascular resistance

Of the 980 patients with available mPAP and PVR data, 538 did not have PH, 223 had PH under the new criteria only, and 219 had PH under the old criteria.

#### Multivariable Cox regression

Multivariable Cox regression analysis demonstrated that compared to the no PH group, there were no statistical differences in waitlist mortality for the new PH group (hazard ratio [HR] 1.37, 95% confidence interval [CI] 0.55-3.40, *p* = 0.50) or the old PH group (HR 0.86, 95% CI 0.20-3.63, *p* = 0.84) (Table 4).

**Table 4:** Multivariable Analysis Examining Effect of Precapillary Pulmonary Hypertension on Hypersensitivity Pneumonitis Transplant Waitlist Mortality

Variable	Multivariable Cox regression
PH status (no PH as reference)	
New precapillary PH	HR 1.37, 95% CI 0.55-3.40, *p* = 0.50
Old precapillary PH	HR 0.86, 95% CI 0.20-3.63, *p* = 0.84
FVC	HR 0.98, 95% CI 0.95-1.01, *p* = 0.24
6MWD^a^	HR 0.98, 95% CI 0.97-0.99, *p* = 0.02
Oxygen requirement^a^	HR 1.20, 95% CI 1.11-1.31, *p* < 0.001
Height	HR 0.97, 05% CI 0.92-1.03, *p* = 0.33
Blood type (A as reference)	
AB	HR 0.91, 95% CI 0.20-4.09, *p* = 0.90
B	HR 0.82, 95% CI 0.16-4.12, *p* = 0.81
O	HR 0.68, 95% CI 0.28-1.64, *p* = 0.39
Male sex	HR 1.96, 95% CI 0.48-8.11, *p* = 0.35
BMI	HR 0.98, 95% CI 0.87-1.09, *p* = 0.66
Age	HR 1.03, 95% CI 0.97-1.09, *p* = 0.36

Abbreviations: 6MWD, 6-minute walk distance; BMI, body mass index; CI, confidence interval; FVC, forced vital capacity; HR, hazard ratio; PH, pulmonary hypertension.

a A variable that has an independent and significant association with transplant waitlist mortality.

### Mean pulmonary artery pressure

Of the 1,029 HP patients listed for lung transplantation with available mPAP data, 455 patients had mPAP ≤20 mm Hg, 240 patients had mPAP >20 to <25 mm Hg, and 334 patients had mPAP ≥25 mm Hg (Figure 1).

#### Kaplan-Meier analysis

**Figure 2:**
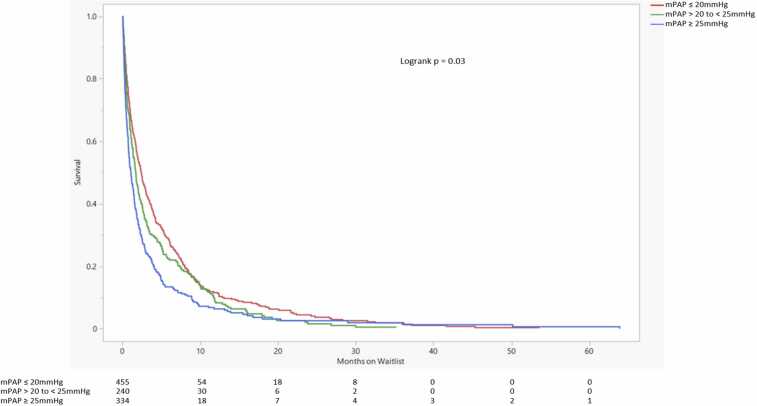
Lung transplant waitlist survival probability comparison. The number of patients at risk of death on the waitlist in each group is displayed at the bottom. Changes in the number of patients attributed to death on the waitlist. Lower waitlist survival probability is seen in the mPAP ≥25 mm Hg group. mPAP, mean pulmonary artery pressure.

**Figure 3:**
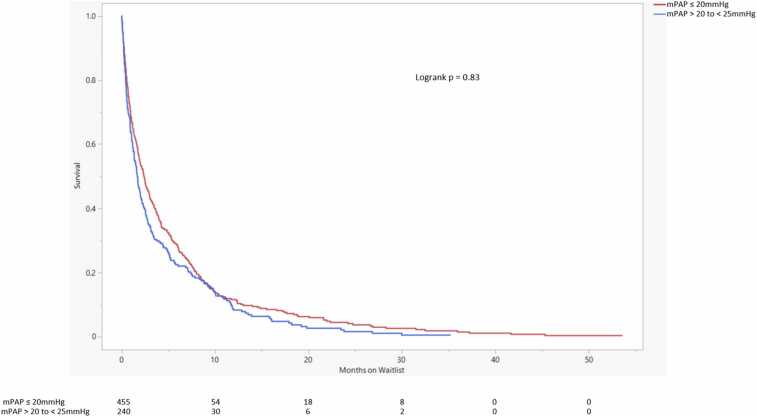
Lung transplant waitlist survival probability comparison. The number of patients at risk of death on the waitlist in each group is displayed at the bottom. Changes in the number of patients attributed to death on the waitlist. There is no significant difference in waitlist survival probability between the 2 mPAP groups. mPAP, mean pulmonary artery pressure.

There was a significant difference in survival probability between the 3 mPAP groups (logrank *p* = 0.03). This difference appeared to be driven by the mPAP ≥25 mm Hg group (Figure 2). Repeat Kaplan-Meier analysis comparing HP patients with mPAP ≤20 mm Hg and HP patients with >20 to <25 mm Hg was performed. There was no significant difference in waitlist survival probability between those 2 groups (logrank *p* = 0.85) (Figure 3).

#### Cox regression

Cox regression showed that mPAP >20 to <25 mm Hg was not associated with increased waitlist mortality when compared to the mPAP ≤20 mm Hg group (HR 0.95, 95% CI 0.62-1.47, *p* = 0.83). However, mPAP ≥25 mm Hg group was significantly associated with increased waitlist mortality when compared to mPAP ≤20 mm Hg (HR 1.57, 95% CI 1.08-2.27, *p* = 0.02) (Table 5).

**Table 5:** Cox Regression Analysis Examining Waitlist Mortality Between Separate Pulmonary Vascular Resistance Groups and Mean Pulmonary Artery Pressure Groups

PH groups	Cox regression	*p*-value
PVR ≤2 WU		
PVR >2 to <3 WU	HR 1.02, 95% CI 0.654-1.576	*p* = 0.95
PVR ≥3 WU	HR 1.64, 95% CI 1.123-2.397	*p* = 0.01
mPAP ≤20 mm Hg		
mPAP >20 to <25 mm Hg	HR 0.95, 95% CI 0.62-1.47	*p* = 0.83
mPAP ≥25 mm Hg	HR 1.57, 95% CI 1.08-2.27	*p* = 0.02

Abbreviations: CI, confidence interval; HR, hazard ratio; mPAP, mean pulmonary artery pressure; PVR, pulmonary vascular resistance; WU, Wood units.

#### Multivariable Cox regression

Multivariable Cox regression showed that, compared to mPAP ≤20 mm Hg, neither mPAP 20 to 25 mm Hg (HR 0.91, 95% CI 0.55-1.48, *p* = 0.69) nor mPAP ≥25 mm Hg (HR 1.32, 95% CI 0.79-2.18, *p* = 0.29) demonstrated significant difference in waitlist mortality when accounting for pertinent variables (Table 6).

**Table 6:** Multivariable Analysis Examining Effect of Separate Pulmonary Vascular Resistance Groups and Mean Pulmonary Artery Pressure Groups on Transplant Waitlist Mortality

Variable	Cox Regression analysis
mPAP grouping (≤20 mm Hg as reference)	
>20 to <25 mm Hg	HR 0.91, 95% CI 0.55-1.48, *p* = 0.69
≥25 mm Hg	HR 1.32, 95% CI 0.79-2.18, *p* = 0.29
PVR grouping (≤2 WU as reference)	
>2 to <3 WU	HR 1.28, 95% CI 0.41-4.01, *p* = 0.67
≥3 WU^a^	HR 2.97, 95% CI 1.179.62, *p* = 0.03
FVC	HR 0.98, 95% CI 0.97-1.11, *p* = 0.35
6MWD^a^	HR 0.98, 95% CI 0.97-0.99, *p* = 0.01
Oxygen requirement^a^	HR 1.20, 95% CI 1.10-1.31, *p* < 0.001
Height	HR 0.99, 95% CI 0.94-1.05, *p* = 0.83
Blood type (type A as reference)	
Type AB	HR 0.84, 95% CI 0.17-4.08, *p* = 0.83
Type B	HR 0.55, 95% CI 0.11-2.84, *p* = 0.48
Type O	HR 0.54, 95% CI 0.22-1.32, *p* = 0.17
Sex (female as reference)	
Male	HR 1.15, 95% CI 0.71-1.87, *p* = 0.56
BMI	HR 0.97, 95% CI 0.93-1.01, *p* = 0.17
Age	HR 1.01, 95% CI 0.99-1.03, *p* = 0.36

Abbreviations: 6MWD, 6-minute walk distance; BMI, body mass index; CI, confidence interval; FVC, forced vital capacity; HR, hazard ratio; mPAP, mean pulmonary artery pressure; PVR, pulmonary vascular resistance; WU, Wood units.

a A variable that has an independent and significant association with transplant waitlist mortality.

### Pulmonary vascular resistance

Of the 1,175 HP patients listed for lung transplantation, 980 had available PVR data. Four hundred and fourteen patients had PVR ≤2 WU, 257 patients had PVR >2 to <3 WU, and 309 patients had PVR ≥3 WU (Figure 1).

#### Kaplan-Meier analysis

**Figure 4:**
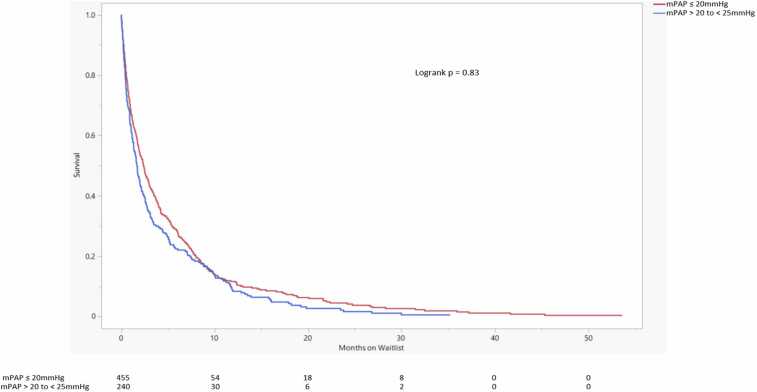
Lung transplant waitlist survival probability comparison. The number of patients at risk of death on the waitlist in each group is displayed at the bottom. Changes in the number of patients attributed to death on the waitlist. Lower waitlist survival probability is seen in the PVR ≥3 WU group. mPAP, mean pulmonary artery pressure; PVR, pulmonary vascular resistance; WU, Wood units.

**Figure 5:**
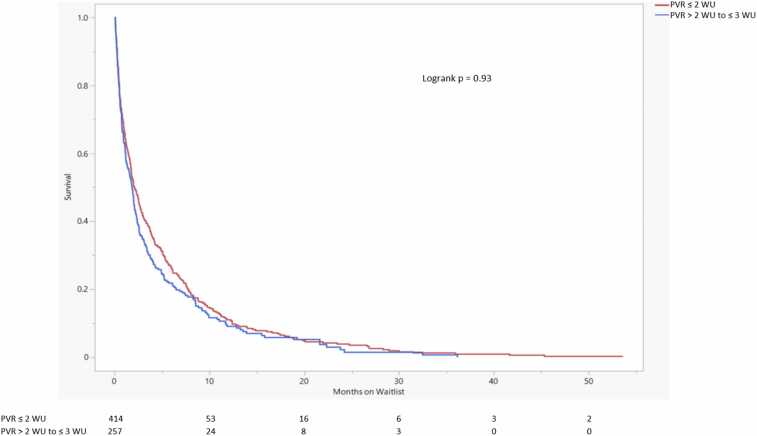
Lung transplant waitlist survival probability comparison. The number of patients at risk of death on the waitlist in each group is displayed at the bottom. Changes in the number of patients attributed to death on the waitlist. There is no significant difference in waitlist survival probability between the 2 PVR groups. PVR, pulmonary vascular resistance; WU, Wood units.

Survival probability between the 3 PVR groups was significantly different, driven mostly by the PVR ≥3 WU group (logrank *p* = 0.02) (Figure 4). Kaplan-Meier survival curves comparing PVR ≤2 WU and PVR >2 to <3 WU did not show a significant difference in waitlist survival probability (logrank *p* = 0.93) (Figure 5).

#### Cox regression

PVR >2 to <3 WU was not associated with increased waitlist mortality when compared to PVR ≤2 WU (HR 1.02, 95% CI 0.654-1.576, *p* = 0.95). When compared to PVR ≤2 WU, PVR ≥3 WU was significantly associated with increased waitlist mortality (HR 1.64, 95% CI 1.123-2.397, *p* = 0.01) (Table 5).

#### Multivariable Cox regression

PVR ≥3 WU demonstrated significantly increased waitlist mortality when compared to PVR ≤2 WU (HR 2.97, 95% CI 1.17-9.62, *p* = 0.03) while accounting for other pertinent variables. There was no significant difference in waitlist mortality comparing PVR >2 to <3 WU with PVR ≤2 WU (HR 1.28, 95% CI 0.41-4.01, *p* = 0.67) (Table 6).

### Other pertinent variables

Multivariable Cox regression demonstrated independent increases in waitlist mortality for 6MWD (HR 0.98, 95% CI 0.97-0.99, *p* = 0.02) and the presence of an oxygen requirement (HR 1.20, 95% CI 1.11-1.31, *p* < 0.001). FVC (HR 0.98, 95% CI 0.95-1.01, *p* = 0.24), height (HR 0.97, 05% CI 0.92-1.03, *p* = 0.33), sex (HR 1.96, 95% CI 0.48-8.11, *p* = 0.35), BMI (HR 0.98, 95% CI 0.87-1.09, *p* = 0.66), and age (HR 1.03, 95% CI 0.97-0.09, *p* = 0.36) were not independently associated with increases in waitlist mortality. Blood types AB (HR 0.91, 95% CI 0.20-4.09, *p* = 0.90), B (HR 0.82, 95% CI 0.16-4.12, *p* = 0.81), and O (HR 0.68, 95% CI 0.28-1.64, *p* = 0.39) did not show a significant increase in waitlist mortality when compared to type A as a baseline (Table 6).

## Discussion

HP lung transplant candidates with precapillary PH determined by the new criteria (mPAP ≥20 mm Hg, PVR ≥2 WU) exhibited no difference in waitlist mortality compared to non-PH patients and PH patients diagnosed via the old criteria. Through individual component analysis, PVR ≥3 WU demonstrated an especially strong correlation with increased waitlist mortality as an HR of 2.6 was calculated even when accounting for additional pertinent variables. There was no increase in waitlist mortality for mPAP ≥25 mm Hg when controlling for other pertinent variables. These results question the effect of the updated ESC PH diagnostic criteria on our population. Our data indicates that a more liberal approach to the diagnosis of PH in HP lung transplant candidates does not identify patients at higher risk for mortality among this specific population. Our analysis demonstrates that PVR ≥3 is a predictor of waitlist mortality, but this effect may be dampened by combining it with mPAP for diagnosis. This suggests that clinicians may benefit from evaluating PVR independently of mPAP when prognosticating HP patients listed for lung transplantation. We also provide an analysis of precapillary PH prevalence in 980 HP patients, the largest analysis to date, reporting a prevalence of 52%.

The lower mPAP and PVR cutoffs may not effectively prognosticate outcomes in our HP cohort for several reasons. The ESC guidelines cite multiple studies influencing their decision to lower diagnostic thresholds. Several of these studies involve invasive measurements of mPAP and PVR in healthy subjects.^9^ Mean values from these studies were used to support “normal” pulmonary vascular hemodynamics and label those with elevated values as having PH. Other referenced studies include large meta-analyses that examine the morbidity and mortality of mild PH, diagnosed by a mix of both invasive and noninvasive methods, for general populations.^10^, ^11^ While patients with lung disease were examined, expected mPAP and PVR values for these patients are not well-established. Our data questions the utility of lowering PVR and mPAP cutoffs for the diagnosis of precapillary PH in ILD without understanding values that represent a true disease state.

Current PH data in HP are limited. One prospective study examined the prevalence of PH in chronic, symptomatic HP using right heart catheterization in a small population of 50 patients. They found a prevalence of 44% using the former criteria of mPAP >25 mm Hg and PVR >3 WU.^12^ Although a small study, the prevalence reported matches a previous retrospective study of 2,525 idiopathic pulmonary fibrosis patients listed for lung transplant which reported a PH prevalence of 44.1%.^13^ To our knowledge, that is the largest prospective study examining PH prevalence in HP patients. Other small case series and retrospective analyses have examined the prevalence of PH in HP but have confounding factors such as noninvasive measurements and patient populations living at high altitudes.^14^, ^15^ Our study provides the largest analysis of precapillary PH prevalence in HP to date. Our reported prevalence of 52% is higher than the above-mentioned studies. This may be due to our population including exclusively lung transplant candidates implying more advanced lung disease. However, our study did control for lung function using FVC, with % pred FVC similar across all mPAP groups and all PVR groups, thus supporting previous data that the presence and severity of PH are not related to the severity of underlying ILD.^16^ Regardless, our study provides further evidence that the prevalence of group 3 PH in HP is significant.

The data regarding outcomes on HP patients with PH specifically are limited. This differs from other ILDs, such as idiopathic pulmonary fibrosis, which have been extensively studied.^17^, ^18^ Data involving group 3 PH in lung transplant candidates is also scarce, but post-transplant outcomes have been studied. Lung transplant recipients with severe parenchymal lung disease and concomitant group 3 PH demonstrate elevated rates of primary graft dysfunction and increased 1-year mortality in patients with severe parenchymal lung disease and concomitant group 3 PH.^19^ One retrospective study in Austria examined mortality in HP patients based on PH. Although they reported a significantly increased mortality rate in HP patients with pulmonary artery systolic pressure (PASP) >35 mm Hg, the study is limited by noninvasive measurements using echocardiography and the use of PASP rather than mPAP or PVR for the analysis.^20^ To our knowledge, there are no other studies examining the outcomes of HP patients with PH, and our study is the largest analysis of PH in HP patients both in terms of prevalence and outcomes. The finding that PVR ≥3 WU was independently associated with increased waitlist mortality is also a novel result. Several studies have demonstrated worse transplantation outcomes associated with the variables used in our multivariable analysis.^21^, ^22^ Although the L-CAS score recognizes PH severity as a factor, the specific value of PVR ≥3 WU has not been recognized as an independent predictor of mortality.^7^ This finding illustrates the importance of PVR in prognostication and should encourage further research on the relationship between PVR and lung transplant outcomes for HP patients.

There are several major strengths of our study. The large sample size allows for the most comprehensive study of PH in HP to date, the study focuses on current gaps in the PH in HP transplant literature, and the statistical analysis accounts for pertinent confounders while providing robust outcome data. Our analysis does have several limitations. The retrospective design and utilization of a general clinical database limit our results. HP is a variable disease with different clinical, radiographic, and histologic subtypes.^1^, ^2^ Each subtype has its own morbidity and mortality.^23^, ^24^, ^25^ The SRTR does not provide information regarding these subtypes that may have an influence on outcomes. In addition, the uniqueness of our population limits extrapolation to larger populations. HP is a rare, unique disease and patients listed for lung transplant are the most severely ill. This limits the utility of our data to a small cohort and prevents extrapolation to both other ILD populations and nontransplant HP patients. Our analysis was also unable to account for the many advancements in PH therapy from 2000-2022 which may have affected outcomes over the broad range of the study.

In conclusion, the new ESC PH diagnostic criteria do not confer increased risk of waitlist mortality for HP patients. When combined, mPAP and PVR are not significantly associated with waitlist mortality. PVR ≥3 demonstrated increased waitlist mortality when evaluated independently from mPAP. This finding persisted when controlling for pertinent variables. The prevalence of precapillary PH in HP patients is also significant and requires further study. Studies involving other ILD populations are necessary to elucidate the effect of the 2022 ESC PH diagnostic criteria on overall ILD outcomes. Evaluation of post-transplant outcomes is also necessary. Identifying risk factors for poor outcomes may lead to changes in pretransplant management, post-transplant management, and better waitlist prognostication. Combining our data with post-transplant outcomes will more comprehensively illustrate the effect of the new ESC PH diagnostic criteria on the HP lung transplantation population.

## CRediT authorship contribution statement

M.J.N., Z.D., and S.G. conceived the study and collected relevant data. M.J.N., Z.D., and S.G. analyzed the data. S.G. provided statistical analysis. M.J.N. drafted the manuscript and all authors contributed substantially to its revision. M.J.N. takes responsibility for the paper as a whole.

## Impact

This study will further our understanding of the implications of more inclusive pulmonary hypertension diagnostic criteria on hypersensitivity pneumonitis lung transplant candidates. It will identify the incidence of pulmonary hypertension in this understudied population. It will call into question the impact of earlier diagnosis on outcomes and encourage further research into this topic for other interstitial lung diseases.

## Disclosure statement

The authors declare that they have no known competing financial interests or personal relationships that could have appeared to influence the work reported in this paper.
